# Molecular Dynamics Study of Polyacrylamide and Polysaccharide-Derived Flocculants Adsorption on Mg(OH)_2_ Surfaces at pH 11

**DOI:** 10.3390/polym17020227

**Published:** 2025-01-17

**Authors:** Gonzalo R. Quezada, Antonia A. Vargas, Steven Nieto, Karien I. García, Pedro Robles, Ricardo I. Jeldres

**Affiliations:** 1 Escuela de Ingeniería Civil Química, Facultad de Ingeniería, Universidad del Bio-Bio, Concepción 4030000, Chile; antonia.vargas2001@alumnos.ubiobio.cl; 2 Advanced Mining Technology Center (AMTC), Universidad de Antofagasta, Antofagasta 1240000, Chile; yeison.nieto.mejia@ua.cl (S.N.); ricardo.jeldres@uantof.cl (R.I.J.); 3 Facultad de Ciencias Ambientales and Centro EULA, Universidad de Concepción, Concepción 4030000, Chile; kariengarcia@udec.cl; 4 Escuela de Ingeniería Química, Pontificia Universidad Católica de Valparaíso, Valparaíso 2340000, Chile; 5 Departamento de Ingeniería Química y Procesos de Minerales, Facultad de Ingeniería, Universidad de Antofagasta, Antofagasta 1240000, Chile

**Keywords:** molecular dynamics, brucite, polysaccharides, polyacrylamide, polymer adsorption, water treatment in mining

## Abstract

Brucite (Mg(OH)_2_) is a typical precipitate in the mining industry that adversely affects processes such as flotation and thickening. Gaining insights into the physicochemical properties of this mineral is critical for developing strategies to mitigate these challenges and improve operational efficiency. Additionally, incorporating natural-origin polymers aligns with the shift toward more sustainable mining practices. In this study, molecular dynamics simulations were employed to investigate the interaction of brucite with polysaccharides such as cellulose, guar gum, and alginate and to compare these with conventional polymers, including polyacrylamide, hydrolyzed polyacrylamide, and polyacrylic acid, under conditions of pH 11 in low-salinity water. The methodology enhanced adsorption sampling by incorporating additional temporary interactions between the polymer and the brucite surface. The results reveal that neutral polymers exhibit stronger and more stable interactions with brucite compared to charged polymers, which is consistent with the neutral nature of brucite under the studied conditions. Van der Waals forces predominantly govern the adsorption of polysaccharides, while Coulombic forces primarily drive interactions involving polyacrylamides. These findings provide valuable insights into the molecular mechanisms of polymer-brucite interactions, facilitating the development of more effective and sustainable mining additives.

## 1. Introduction

Mineral processing in arid and semi-arid regions faces a critical challenge due to the limited availability of water resources, as mineral concentration operations are highly dependent on access to water. To address this issue, many large-scale mining companies have used low-quality water as an alternative, including the direct use of seawater or its use after a desalination process [1,2]. While this strategy contributes to alleviating the pressure on inland water resources, its implementation involves significant costs since deposits are often located hundreds of kilometers from the coast and at high altitudes, considerably increasing the costs associated with water transport. In this context, water recovery in stages such as tailings thickening takes on a crucial role, as efficient recovery decreases freshwater consumption and reduces operational costs related to the use of seawater. However, this alternative presents significant technical challenges, particularly in processes like thickening, where tailings from flotation are mixed with high molecular weight flocculants to accelerate sedimentation and maximize water recycling [3,4].

During the tailings thickening process, pulps are treated with high molecular weight soluble polymers, known as flocculants, which promote particle aggregation and the formation of flocs with a high sedimentation rate. The efficiency of this stage depends on various factors, including the material’s mineralogy, the process’s technological characteristics, the operational management of the thickener, and the quality of the water used. In this context, the presence of salts plays a determining role, generating significant changes in the physicochemical interactions between the flocculants and mineral surfaces, which can directly influence process performance [5,6,7,8].

Under saline conditions, cationic bridges are formed between functional groups of the polymer and the particle surface, which favors the adsorption of the polymer. However, traditional anionic polymers, such as partially hydrolyzed polyacrylamide (HPAM), tend to coil in the presence of divalent ions such as calcium and magnesium, which reduces their radius of gyration and impairs their ability to form effective polymeric bridges between particles. Quezada et al. [9] analyzed the water-quartz interface in the presence of HPAM under low and high salinity conditions using molecular dynamics simulations. Their findings reveal that, in salt water, the adsorption between HPAM and quartz significantly increases due to the formation of salt bridges. In contrast, such adsorption is limited to a few contacts between surfaces in fresh water. In this context, molecular dynamics simulations have emerged as a crucial tool for understanding in detail the mechanisms of interaction between flocculants and mineral particles [10,11,12,13,14].

Sedimentation can also be influenced by the formation of complexes and colloidal precipitates, such as brucite (Mg(OH)_2_). According to Ramos et al. [15], Mg(OH)_2_ precipitates decrease the flocculation efficiency of quartz-kaolin clayey tailings by competing with the mineral surface for the adsorption of the anionic flocculant HPAM (partially hydrolyzed polyacrylamide), which significantly reduces its performance. On the other hand, through molecular dynamics simulations, Quezada et al. [16] demonstrated that magnesium precipitates interfere with the interaction between HPAM and the surfaces of quartz and kaolin particles. This interference decreases the adsorption of the flocculant on the tailings and limits the formation of effective bridges between particles. Furthermore, they indicated that the adsorption of HPAM on the brucite surface occurs mainly through the interaction between the deprotonated oxygen of the acrylic group of the polymer and the hydroxide oxygen of the brucite, with an additional contribution from hydrogen bonds between the nitrogen of the acrylamide group and the hydroxide oxygen.

For their part, Nieto et al. [17] examined the effects of seawater and lime-treated seawater to reduce the Mg content to pH 11 on the consolidation of flocculated quartz-kaolin suspensions using HPAM as a flocculant. Their results showed that, in the presence of seawater, more compact, rigid, and deformation-resistant aggregates formed, making it challenging to release trapped water during the consolidation stage. This phenomenon is attributed to a little-studied adhesive effect of Mg precipitates, which strengthens flocculated suspensions in seawater compared to treated seawater.

An important strategy to optimize the thickening process lies in improving reagent management, considering two key aspects: (i) that the implemented changes do not require significant interventions or additional investments in the process, and (ii) that reagent management has a significant impact on the operation since adjustments in dosage, injection points or reagent types can generate relevant improvements in process efficiency. For example, Grabsch et al. [18] studied calcite flocculation using two commercial flocculants, Magnafloc^®^ 336 and Rheomax^®^ DR1050, observing significant differences in their performance depending on the operating conditions. Magnafloc^®^ showed greater efficiency at low solids concentrations (20–40 kg/m^3^), while Rheomax^®^ produced larger aggregates and better sedimentation rates at higher solids concentrations (≥80 kg/m^3^). These differences are attributed to the higher fractal dimension and denser structures formed in the presence of Rheomax^®^ DR1050, making it particularly suitable for thickening applications in feeds with high solids contents.

Using molecular dynamics simulations, Quezada et al. [19] studied the interactions between polyacrylamide (PAM), HPAM, polyacrylamide-co-sulfonate (PAMPS), poliacrilate acid (PAA), polyethylene oxide (PEO), and guar gum polymers with the kaolinite surface in low and high salinity media. The study identified that PAM and guar gum show a higher affinity with kaolinite under low salinity conditions, while HPAM is more effective in environments with high salt concentrations. The results revealed that the predominant interactions occur through cationic bridges and hydrogen bonds and that the polymer conformations depend significantly on the salinity of the medium.

The growing interest in biodegradable flocculants based on polysaccharides [20,21,22,23], such as chitosan, cellulose, and alginate, strengthens the relevance of combining experimental studies and molecular simulations. Conzatti et al. [24] improved the flocculation properties of chitosan films through PNIPAM grafting using plasma and UV polymerization. Ren et al. [25] developed a thermoresistant flocculant based on chitosan, effective in separating contaminants like tetracycline and Cu(II), with regeneration capability. Wang et al. [26] created aerogels of carboxylated cellulose nanofibers, showing high efficiency in flocculation and water absorption. Additionally, Vijayalakshmi et al. [27] used hybrid beads of nanochitosan and cellulose to adsorb metal ions like Pb(II), while Tian et al. [28] synthesized modified alginate flocculants effective in removing heavy metal ions. These studies highlight natural polymers as sustainable alternatives to synthetic ones in applications requiring efficiency under challenging conditions.

These results raise an interesting line of research: how do flocculants with different physicochemical properties interact with magnesium precipitate surfaces? This approach seeks to identify a reagent capable of improving the sedimentation of tailings in low-quality waters. The present research is carried out through molecular dynamics simulations, allowing detailed control of the flocculant characteristics and an in-depth analysis of the interactions of its functional groups with the surface of brucite, the main precipitate formed from magnesium. While real flocculants typically exhibit molecular weights exceeding 10^6^ Da, the polymers simulated in this study are significantly smaller due to computational constraints. This approach focuses on elucidating the fundamental mechanisms of interaction at a nanometric scale, providing critical insights into the behavior of flocculants under saline conditions. The reagents selected for this study include guar gum, cellulose, alginate, PAM, HPAM, and PAA. This investigation allows the identification of the functional groups present in the polymers that have a lower affinity with the surface of brucite, which could be advantageous for optimizing the sedimentation of clayey tailings generated in mining operations. The manuscript is structured as follows: Section 2 describes the methodology and simulation setup, Section 3 presents the results, and Section 4 discusses the findings and their potential applications.

## 2. Materials and Methods

### 2.1. Compounds

**Figure 1:**
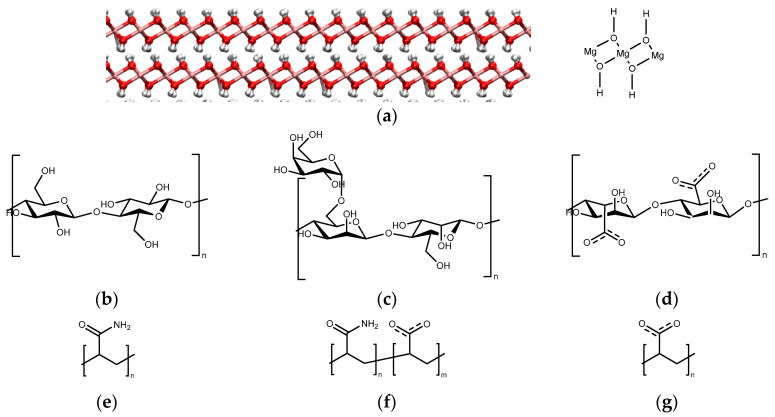
Structure of System Components. (**a**) Brucite (001) surface red spheres are oxygen; pink spheres are magnesium, and white spheres are hydrogen; monomer structures of the polymers studied (**b**) cellulose (**c**) guar gum (**d**) alginate (**e**) PAM (**f**) HPAM and (**g**) PAA.

This study focused on brucite’s basal face (001) using the structure developed in [16], which consists of layered sheets with magnesium bonded to six hydroxide groups, forming an octahedral structure. At the same time, the oxygens have three bonds to different magnesium (Figure 1a). This structure was formed using a unit cell of (a, b, c) = (0.5540, 0.3205, 0.4840), (α, β, γ) = (90, 90, 90), from which a supercell of 20 × 32 × 2 was generated, yielding a surface area of 113.63 nm² and a thickness of 0.90 nm. The polymers studied in this work included six polymers: three from the polysaccharide family and three from the polyacrylamide family (Figure 1b). From the polysaccharide family, the studied polymers were cellulose, alginate, and guar gum. From the polyacrylamide family, the studied polymers were neutral PAM, HPAM, and PAA. For the polysaccharides, the three studied cases are described as follows: cellulose consists of D-glucose units linked by (1→4) bonds [29], alginate is a copolymer where β-D-mannuronate (M) and α-L-guluronate (G) units alternate and are linked by (1→4) bonds [30], and guar gum consists of linear α-D-galactose units linked by (1→4) bonds, with one of the units laterally connected to a β-D-mannose unit through a (1→6) bond, forming hanging groups [31]. The polyacrylamide family consists of neutral polyacrylamide, which consists only of acrylamide monomers; HPAM, consisting of 25% acrylate groups and 75% acrylamide groups arranged every three acrylamide units with one acrylate; and PAA, that consists solely of acrylate groups. The monomers used were 12 for polysaccharides and 48 for polyacrylamides, giving a similar linear length of 12 nm. The pH for investigating these systems was pH 11, which means that brucite is in its neutral state or isoelectric point. Cellulose, guar gum, and PAM maintain neutrality, while alginate, HPAM, and PAA have fully charged negative groups due to their pKa values ranging from 4 to 5. The ions present, such as sodium and chlorine, are represented by ionic atomic units dissolved in the medium. Water molecules are represented as one oxygen atom and two hydrogen atoms.

### 2.2. Forcefield

Brucite, or Mg(OH)_2_, was used with the CLAYFF force field [32,33], which has been able to represent many compounds such as clays, hydroxides, and hydroxyoxides. Its flexibility in modeling nonbonded and intra-layer interactions makes it particularly suitable for adsorption studies. Since its structure is neutral at the studied pH, including charge corrections was unnecessary. This mainly involves constructing the molecules with Avogadro (version 1.2.0) and then generating the topology with Antechamber (version 22.0) [34,35] under the GAFF force field, which provides reliable parameters for diverse functional groups common in organic molecules. The present ions were modeled using the parameters from Li et al. [36] chosen for the SPC/E water model [37] with SETTLE [38] to model the water molecule dynamics. MD simulations were run in Gromacs (version 2022.1) [39], where files from Antechamber were transformed into Gromacs format using the ACPYPE program (version 2022.6.6) [40]. This workflow, validated by previous studies [41,42], combines accuracy and efficiency, aligning with recent methodologies applied in similar polymer-surface interaction studies.

### 2.3. Initial Configuration

The initial configurations were built using a custom code to place each element in the system at a chosen location. First, the size of the simulation box was defined from the brucite crystal, which gave dimensions of 11.08 × 10.25 × 10.00 nm^3^. The surface was placed on the lower xy-plane, and the thickness of the brucite is 0.9 nm with an area of 113 nm^2^, resulting in an available fluid volume of approximately 1000 nm^3^. The polymer was initially placed on the surface with its center of mass located at a standard distance of 3 nm from the surface. In the beginning, the polymer was configured linearly with a random orientation to avoid proximity to its periodic image. Then, the ions were placed randomly with a minimum distance of 0.5 nm between all compounds. Next, water was inserted into the simulation box from a previously equilibrated configuration at 300 K. Water molecules less than 0.2 nm away from other compounds were removed from the final configuration. Finally, to consider only the brucite surface that the polymer will interact with, the simulation box was extended in the z-direction by 15 nm of space, creating a simulation box of 11.08 × 10.25 × 25.00 nm^3^.

### 2.4. Molecular Dynamics

**Figure 2:**
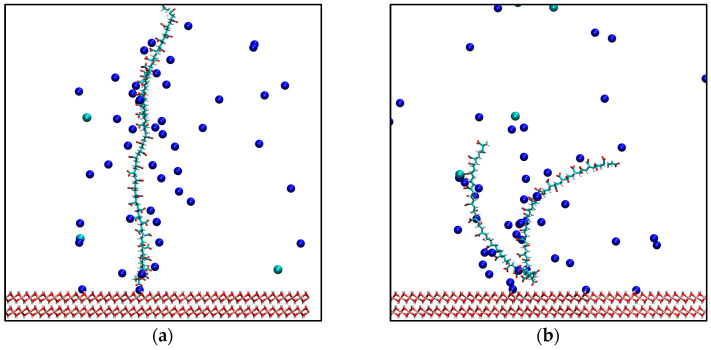
Prepared configurations for sample the interactions with the surface: (**a**) End-grafted configuration; (**b**) U-shaped configuration. Green spheres: chloride ions, blue sphere: sodium ions.

The molecular dynamics simulation strategy was developed based on the results of previous works [19], which provided evidence that adsorption sampling through MD is slow and challenging to capture. A new approach was used to force the polymer to be grafted onto the surface to optimize time and improve the sampling of polymer adsorption on the surface. To achieve this, fictitious interactions were generated between the surface and the polymer to guide the polymer into two study configurations: the first, where one end of the chain adsorbs in a perpendicular form denominated “End-grafted” configuration (Figure 2a), and the second, where a central group of the molecule adsorbs denominated “U-shaped” configuration (Figure 2b).

**Figure 3:**
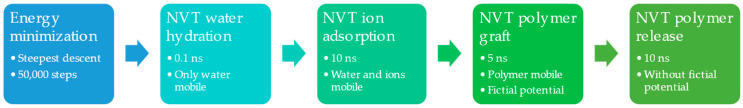
Flow diagram of the molecular dynamics simulations.

The simulation procedure is shown in Figure 3; it began with an energy minimization step to reduce the likelihood of simulation failure due to the generated initial configuration. This was followed by an NVT ensemble simulation where only the water molecules can move to form hydration layers around all the present compounds. Next, an NVT simulation was performed where water and ions could move to generate ion adsorption on the surface and the polymer. Then, an NVT simulation was carried out where fictitious interactions were activated to bring the polymer closer to the surface and keep it in that position. Finally, a final NVT simulation is conducted where all interactions are released, and the stability of the interactions between the polymer and the surface is analyzed.

The integration time step was 2 × 10^−6^ ns, which, together with the restrictions of HBs by LINCS [43], is suitable for the simulations as it does not produce artifacts or energy losses. Temperature and pressure controls were applied using the Nose-Hoover thermostat [44,45] with a relaxation time of 0.0025 ns and the isotropic Parrinello-Rahman barostat [46] with a relaxation time of 0.001 ns. The cut-off radii for van der Waals and Coulombic energies were set to 1.2 nm. Long-range corrections were applied using the Ewald particle mesh method [47]. Crossed interactions in LJ energy were defined using Lorentz-Berthelot’s mixing rules, where COO···Na interactions were corrected by the work of Yoo and Aksimentiev [48]. All simulations were performed in triplicate.

In the NVT step with fictitious interaction, the minimum interaction between the surface and the polymer was measured to confirm that the simulation leads to an interaction suitable for forming interactions in the next step. Then, in the final simulation (NVT polymer release), the duration and number of contacts generated between the polymer and the surface were analyzed. Hydrogen bonds were analyzed using the Gromacs code, and cationic bridges were analyzed using a custom Python code.

## 3. Results and Discussion

### 3.1. Polymer Approach to the Surface

**Figure 4:**
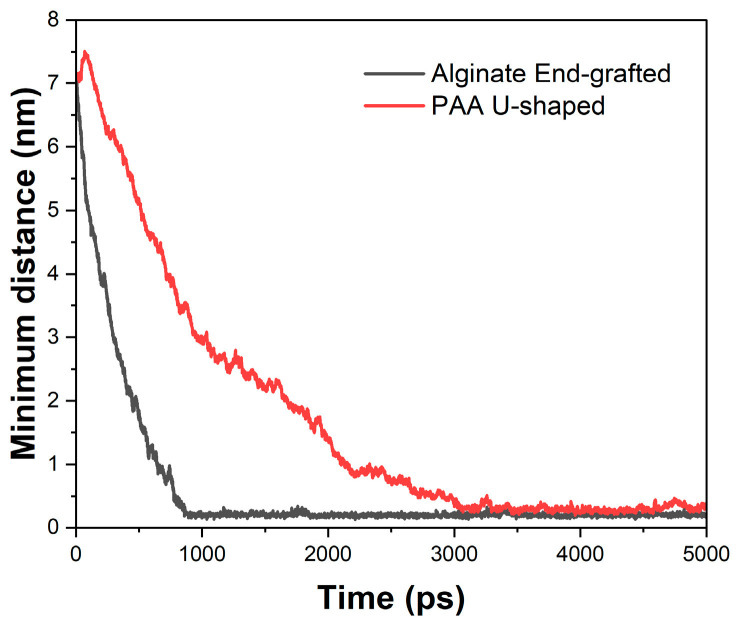
Minimum distances between polymers and the brucite surface in time.

This section analyzes the approach of bringing the polymer closer to the brucite surface under the additional interactions added to the simulation. The study was conducted for all polymers in both U-shaped and end-grafted configurations (Figure 2). In this case, only one example for each configuration is presented to illustrate how the approach works. Figure 4 presents two example configurations: one for alginate in an end-grafted configuration and another for PAA in a U-shaped configuration. The graph illustrates that the alginate in the end-grafted configuration reaches the surface quickly, within approximately 1000 ps. In contrast, the u-shaped configuration for PAA takes considerably longer, around 3000 ps, to establish its contact with the surface. This difference arises from the adopted configurations: positioning a polymer’s end onto the surface is simpler because it requires less structural deformation. Conversely, the u-shaped configuration requires bending the polymer chain to enable the central monomer to interact with the surface, which naturally extends the timescale. Notably, the velocities at which these interactions occur are within the range expected for molecular dynamics simulations and do not induce significant perturbations to the system. The results also confirm that a simulation time of 5 ns is sufficient to generate these configurations. Even in the slowest cases, such as PAA in the u-shaped configuration, stable interactions with the surface were achieved within this timeframe. This finding validates the methodology used to study polymer-surface interactions.

### 3.2. Polymer Interactions with a Brucite Surface

The number of interactions between the polymer and the brucite surface was measured, considering all polymer atoms—carbon, hydrogen, oxygen, and nitrogen—within a cut-off radius of 0.5 nm. These measurements were taken immediately after the fictitious interactions were released for both u-shaped and end-grafted configurations, allowing the polymers to interact freely with the surface.

**Figure 5:**
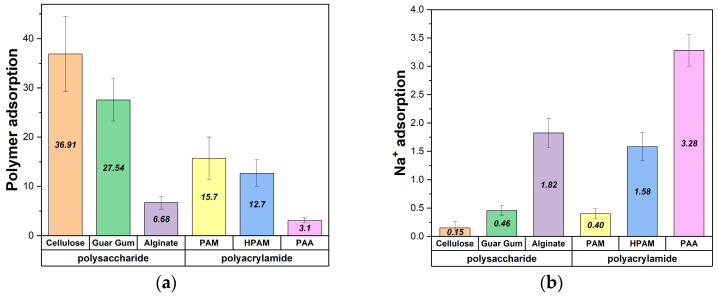
(**a**) Average polymer-surface interactions within a 0.5 nm cut-off radius. (**b**) Cation-surface interactions enhancing adsorption of charged polymers at 0.006 M NaCl.

The results, shown in Figure 5a, reveal that neutral polymers such as cellulose, guar gum, and PAM exhibit a higher average number of interactions with the brucite surface. This trend is consistent with the surface’s overall neutral character. However, at a local scale, the brucite surface is highly anionic due to its adsorption of cations, which facilitates interactions with neutral groups.

Interestingly, PAM shows slightly more interactions than HPAM, although the difference is not pronounced. This could be attributed to the partial hydrolysis of HPAM, introducing negatively charged acrylate groups that may experience mild electrostatic repulsion from the anionic regions of the brucite surface. However, the difference is mitigated by sodium ions (Figure 5b), which act as mediators and enable HPAM to maintain a comparable level of adsorption.

In contrast, strongly charged polymers like alginate and PAA exhibit fewer direct interactions with the surface. As shown in Figure 5b, cations play a critical role in mediating the adsorption of these charged polymers. Although all systems were simulated at a sodium chloride concentration of 0.006 M, charged polymers inherently carry additional counterions, which help to neutralize the system and enhance their adsorption onto the brucite surface despite their lower direct affinity.

**Figure 6:**
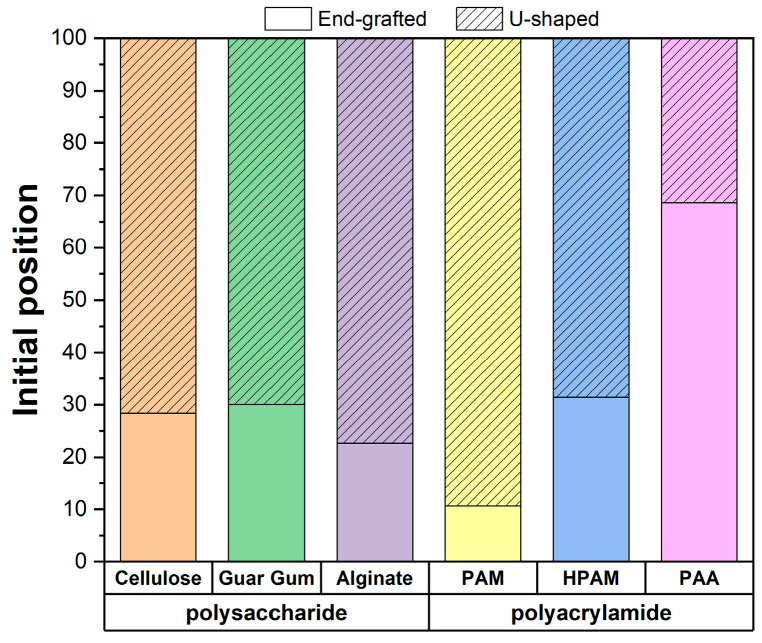
Proportion of end-grafted and u-shaped polymer interactions.

Figure 6 shows the proportion of polymer-surface interactions as a function of the initial polymer configuration (end-grafted and u-shaped). The results indicate that the end-grafted configuration accounts for 28% of cellulose, 30% in guar gum, 23% in alginate, 10% in PAM, 30% in HPAM, and 70% in PAA. These findings suggest that for polysaccharides, the proportion of end-grafted interactions is quite similar between guar gum and cellulose, while it is notably lower for alginate. In contrast, the polyacrylamide family increasingly prefers the end-grafted configuration as the polymer’s charge increases. This trend is due to the general repulsion between the charged polymers and the brucite surface, which is locally anionic. The pronounced preference for the end-grafted position in PAA can be attributed to its higher charge density than alginate, further emphasizing the electrostatic repulsion with the brucite surface.

### 3.3. Hydrogen Bonds and Cationic Bridges

Hydrogen bonds and cationic bridges were analyzed to better understand the adsorption mechanisms of the polymers on the brucite surface. Hydrogen bonds were defined as interactions between the polymer and the surface where the donor-acceptor distance was below 0.35 nm. Cationic bridges were defined as configurations where a sodium ion simultaneously interacts with an oxygen atom from the surface and an oxygen atom from the polymer when the distance was below 0.30 nm in both cases.

**Figure 7:**
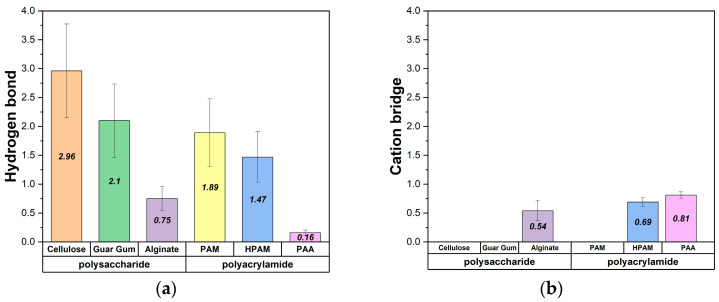
(**a**) Average number of hydrogen bonds formed between polymers and the brucite surface. (**b**) Average number of cationic bridges.

The results, shown in Figure 7, indicate that neutral polymers (cellulose, guar gum, and PAM) form the highest number of hydrogen bonds, highlighting hydrogen bonding as the primary mechanism driving their adsorption onto the surface. This behavior is consistent with the chemical composition of these polymers, which possess functional groups such as hydroxyl or amide groups capable of forming strong hydrogen bonds with surface hydroxyl groups. These results align with the expected behavior of the polymers based on their charge properties. Neutral polymers (cellulose, guar gum, and PAM) exhibit stronger adsorption to the surface, as previously discussed, and form the highest number of hydrogen bonds. This indicates that hydrogen bonding is the primary mechanism driving their adsorption. The lack of electrostatic repulsion further enhances the strength and stability of these interactions, allowing the polymers to form more extensive contact with the brucite surface.

In contrast, charged polymers (alginate, HPAM, and PAA) form fewer hydrogen bonds due to electrostatic repulsion from the anionic nature of the brucite surface. However, their adsorption is facilitated by forming cationic bridges, where sodium ions act as mediators between the polymer and the surface. These interactions provide an alternative adsorption pathway, overcoming repulsive forces to establish localized electrostatic attractions. The relatively low number of cationic bridges can be attributed to the low concentration of ions or salts in the system, limiting the availability of sodium ions to mediate these interactions. This limitation suggests that increasing ionic strength in the system could enhance the adsorption of charged polymers by promoting the formation of additional cationic bridges.

### 3.4. Interaction Energies Between Polymers and Brucite

The interaction energies between the polymers and the brucite surface were calculated, with short-range contributions analyzed separately as Coulombic and Lennard-Jones (LJ) energies. Although the Coulombic contributions from PME cannot be explicitly decomposed to isolate only the polymer-surface interactions, the results offer valuable comparative insights across the studied cases, providing a meaningful perspective even without capturing the full interaction energies.

**Figure 8:**
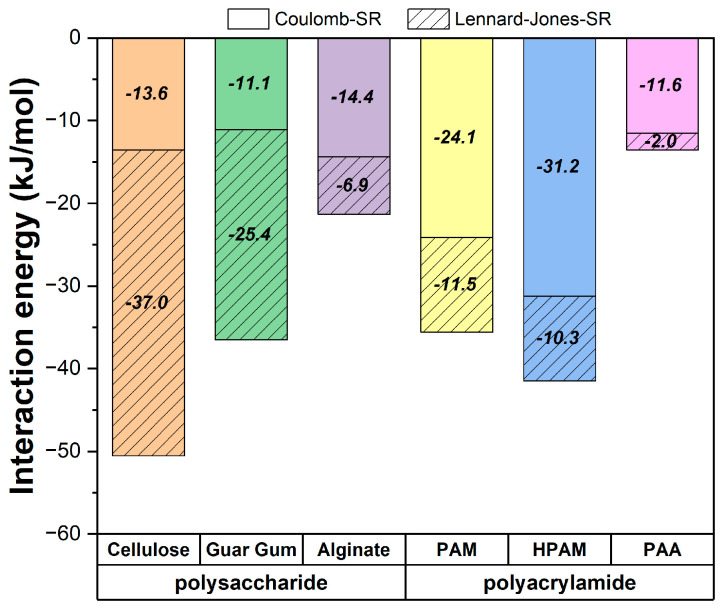
Proportions of van der Waals and Coulomb interaction energies contributing to the total interaction energy.

The results in Figure 8 reveal a trend that aligns with the previous adsorption analyses but highlights differences in the dominant interaction types depending on the polymer. For polysaccharides (cellulose, guar gum, and alginate), the interactions are predominantly governed by Lennard-Jones forces. This suggests that van der Waals interactions, likely involving the aromatic-like rings in the polysaccharide structures, play a significant role. These rings allow for close surface contacts that enhance the LJ contributions and minor Coulombic interactions.

In contrast, for polyacrylamides (PAM, HPAM, and PAA), Coulombic interactions dominate, reflecting the role of their charged or partially charged acrylamide and acrylate groups. Unlike polysaccharides, the main chain of polyacrylamides does not interact directly with the surface. Instead, their adsorption relies on specific interactions, such as hydrogen bonds or cationic bridges, driven by the charges on their functional groups. The stronger Coulomb contributions observed in charged polymers like HPAM and PAA further emphasize this dependence on electrostatic forces.

The distinct contributions from LJ and Coulombic interactions highlight the interplay between the polymer structure and the brucite surface. Structural flexibility and surface contacts are key for polysaccharides, while for polyacrylamides, the presence of charged groups and their ability to form electrostatic interactions determine the adsorption behavior.

### 3.5. Stable Configurations and Interaction Analysis

**Figure 9:**
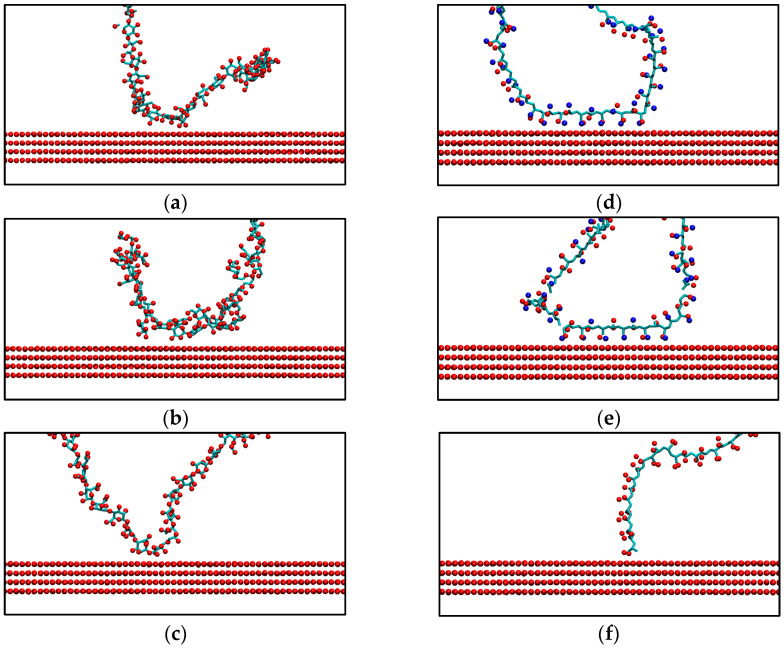
Stable configurations of polymers on the brucite surface: (**a**) Cellulose, (**b**) Guar Gum, (**c**) Alginate, (**d**) PAM, (**e**) HPAM, and (**f**) PAA. Red sphere are oxygen atoms, blue sphere are nitrogen atoms and cyan tubes are the carbon atoms.

The most stable configurations identified during the simulations were visualized to analyze polymer-surface interactions (Figure 9). The predominant configuration across most polymers was the u-shape, while the end-grafted configuration was observed only for PAA. For cellulose, approximately eight oxygen atoms were found to interact closely with the brucite surface. This behavior highlights the structural adaptability of cellulose and its ability to maximize interactions through its numerous hydroxyl groups. Similarly, guar gum exhibits around five oxygen atoms interacting with the surface, while alginate shows three such interactions. The proximity of the pyranose rings in polysaccharides, such as cellulose and guar gum, to the surface indicates that their interactions are predominantly Lennard-Jones (LJ) in nature. This is because the hydrogen atoms in the pyranose rings, with partial charges ranging from +0.06*e* to +0.10*e*, have relatively low interaction strength compared to hydroxyl or carboxylic groups.

In the case of polyacrylamides, the images reveal that for PAM, several acrylamide side groups establish interactions with the surface, leading to Coulombic interactions mediated by the brucite’s local charges. These interactions are primarily driven by the amine and carbonyl groups, which exhibit partial charges between −0.6*e* and −0.8*e*. The high magnitude of these partial charges enhances the strength of the interactions with the brucite surface, facilitating adsorption. A similar phenomenon is observed with HPAM, where acrylamide groups contribute to the interactions. However, its charged acrylate groups do not appear to interact directly with the surface. Finally, the most stable configuration for PAA shows that only a terminal acrylate group, along with its CH_3_ group, anchors to the surface. This behavior demonstrates the minimal interaction sites for this polymer and highlights the specific structural arrangement that stabilizes its adsorption. In contrast, the U-shape configuration was transient and not stable, reinforcing that the end-grafted configuration is the primary adsorption mode for PAA.

## 4. Conclusions

This study uses a novel methodology in molecular dynamics simulations, where forced configurations (end-grafted and u-shaped) were used to improve the sampling of polymer adsorption on surfaces. This approach addressed the limitations of traditional methods, where adsorption events were slower and more challenging to analyze. The forced configurations allowed for the efficient identification of stable polymer-surface interactions, with the u-shaped configuration emerging as predominant. These advancements accelerated the sampling process and provided clearer insights into the adsorption mechanisms. Although the simulated polymers are smaller than those used in industry, the results offer a foundational understanding of nanometric-scale adsorption mechanisms, essential for advancing flocculation studies at larger scales.

From a scientific perspective, the findings reveal how polymers’ structural and chemical properties influence their adsorption behavior on surfaces with mixed characteristics, such as brucite. Polysaccharides, including cellulose and guar gum, exhibited strong adsorption driven primarily by Lennard-Jones interactions, facilitated by their hydroxyl-rich structures and pyranose rings. In contrast, polyacrylamides, such as PAM and HPAM, relied on Coulombic interactions mediated by their acrylamide or acrylate groups, with adsorption largely influenced by electrostatic forces and local charge distributions. In terms of practical applications, these results highlight the potential for designing hybrid polymers that combine polysaccharides’ structural flexibility with polyacrylamides’ electrostatic properties. Such materials could enhance adsorption efficiency in industrial processes like tailings thickening and water recovery. Future studies will explore higher salt concentrations, providing further insights into the role of cationic bridges and optimizing polymer designs for industrial applications.

## Data Availability

Dataset available on request from the authors.

## Abbreviations

The following abbreviations are used in this manuscript:

Mg(OH)_2_	Magnesium hydroxide/Brucite
PAM	Polyacrylamide
HPAM	Hydrolyzed polyacrylamide
PAA	Polyacrylic acid
PAMPS	Polyacrilamide-co-propanesulfonic
PEO	Polyethylene oxide
PNIPAM	Poly-N-isopropylacrylamide
UV	Ultraviolet
Da	Dalton [g/mol]
CLAYFF	Clay force field
GAFF	General Amber force field
MD	Molecular Dynamics
NVT	Constant number of molecules, volume and temperature
LJ	Lennard-Jones

## References

[1] Jeldres R.I., Nieto S., Jeldres M. (2021). Manejo de Relaves Mineros En Agua de Mar. Economía Circular en Procesos Mineros.

[2] Cisternas L.A., Gálvez E.D. (2018). The Use of Seawater in Mining. Miner. Process. Extr. Metall. Rev..

[3] Moody G.M. (2007). Polymeric Flocculants. Handbook of Industrial Water Soluble Polymers.

[4] Hogg R. (2013). Bridging Flocculation by Polymers. KONA Powder Part. J..

[5] Mpofu P., Addai-Mensah J., Ralston J. (2003). Influence of Hydrolyzable Metal Ions on the Interfacial Chemistry, Particle Interactions, and Dewatering Behavior of Kaolinite Dispersions. J. Colloid Interface Sci..

[6] Cruz N., Peng Y., Wightman E., Xu N. (2015). The Interaction of Clay Minerals with Gypsum and Its Effects on Copper–Gold Flotation. Miner. Eng..

[7] Costine A., Cox J., Travaglini S., Lubansky A., Fawell P., Misslitz H. (2018). Variations in the Molecular Weight Response of Anionic Polyacrylamides under Different Flocculation Conditions. Chem. Eng. Sci..

[8] Peng F.F., Di P. (1994). Effect of Multivalent Salts—Calcium and Aluminum on the Flocculation of Kaolin Suspension with Anionic Polyacrylamide. J. Colloid Interface Sci..

[9] Quezada G.R., Rozas R.E., Toledo P.G. (2021). Polyacrylamide Adsorption on (1 0 1) Quartz Surfaces in Saltwater for a Range of PH Values by Molecular Dynamics Simulations. Miner. Eng..

[10] Ren B., Xu M., Zhu J., Xia Z., Liu C., Min F. (2025). Experimental and MD Simulation of the Hydrophobic Adsorption Mechanism of P(AM-MAPTAC-TEMAc6) at Kaolinite/Water Interface. Appl. Surf. Sci..

[11] Zhang X., Zhao Y., Zhang Z., Wang S. (2021). Investigation of the Interaction between Xanthate and Kaolinite Based on Experiments, Molecular Dynamics Simulation, and Density Functional Theory. J. Mol. Liq..

[12] Chang Z., Sun C., Kou J., Fu G., Qi X. (2021). Experimental and Molecular Dynamics Simulation Study on the Effect of Polyacrylamide on Bauxite Flotation. Miner. Eng..

[13] Zhang J., Yang C., Niu F., Gao S. (2022). Molecular Dynamics Study on Selective Flotation of Hematite with Sodium Oleate Collector and Starch-Acrylamide Flocculant. Appl. Surf. Sci..

[14] He Z., Wang J., Liao B., Bai Y., Shao Z., Huang X., Wang Q., Li Y. (2022). Molecular Simulation of Interactions between High-Molecular-Polymer Flocculation Gel for Oil-Based Drilling Fluid and Clay Minerals. Gels.

[15] Ramos J.J., Leiva W.H., Castillo C.N., Ihle C.F., Fawell P.D., Jeldres R.I. (2020). Seawater Flocculation of Clay-Based Mining Tailings: Impact of Calcium and Magnesium Precipitation. Miner. Eng..

[16] Quezada G.R., Jeldres M., Toro N., Robles P., Toledo P.G., Jeldres R.I. (2021). Understanding the Flocculation Mechanism of Quartz and Kaolinite with Polyacrylamide in Seawater: A Molecular Dynamics Approach. Colloids Surfaces A Physicochem. Eng. Asp..

[17] Nieto S., Toledo P.G., Robles P., Quezada G.R., Jeldres R.I. (2023). Impact of Magnesium on the Flocculation, Sedimentation and Consolidation of Clay-Rich Tailings in Lime-Treated Seawater. Sep. Purif. Technol..

[18] Grabsch A.F., Fawell P.D., Adkins S.J., Beveridge A. (2013). The Impact of Achieving a Higher Aggregate Density on Polymer-Bridging Flocculation. Int. J. Miner. Process..

[19] Quezada G.R., Leiva W., Saavedra J.H., Robles P., Gálvez E., Jeldres R.I. (2022). A Molecular Dynamics Simulation of Polymers’ Interactions with Kaolinite (0 1 0) Surfaces in Saline Solutions. Polymers.

[20] Salehizadeh H., Yan N., Farnood R. (2018). Recent Advances in Polysaccharide Bio-Based Flocculants. Biotechnol. Adv..

[21] Fauzani D., Notodarmojo S., Handajani M., Helmy Q., Kardiansyah T. (2021). Cellulose in Natural Flocculant Applications: A Review. J. Phys. Conf. Ser..

[22] Sharma B.R., Dhuldhoya N.C., Merchant U.C. (2006). Flocculants—An Ecofriendly Approach. J. Polym. Environ..

[23] Lee C.S., Robinson J., Chong M.F. (2014). A Review on Application of Flocculants in Wastewater Treatment. Process Saf. Environ. Prot..

[24] Conzatti G., Chamary S., De Geyter N., Cavalie S., Morent R., Tourrette A. (2018). Surface Functionalization of Plasticized Chitosan Films through PNIPAM Grafting via UV and Plasma Graft Polymerization. Eur. Polym. J..

[25] Ren K., Du H., Yang Z., Tian Z., Zhang X., Yang W., Chen J. (2017). Separation and Sequential Recovery of Tetracycline and Cu(II) from Water Using Reusable Thermoresponsive Chitosan-Based Flocculant. ACS Appl. Mater. Interfaces.

[26] Wang D., Yu H., Fan X., Gu J., Ye S., Yao J., Ni Q. (2018). High Aspect Ratio Carboxylated Cellulose Nanofibers Cross-Linked to Robust Aerogels for Superabsorption-Flocculants: Paving Way from Nanoscale to Macroscale. ACS Appl. Mater. Interfaces.

[27] Vijayalakshmi K., Devi B.M., Latha S., Gomathi T., Sudha P.N., Venkatesan J., Anil S. (2017). Batch Adsorption and Desorption Studies on the Removal of Lead (II) from Aqueous Solution Using Nanochitosan/Sodium Alginate/Microcrystalline Cellulose Beads. Int. J. Biol. Macromol..

[28] Tian Z., Zhang L., Shi G., Sang X., Ni C. (2018). The Synthesis of Modified Alginate Flocculants and Their Properties for Removing Heavy Metal Ions of Wastewater. J. Appl. Polym. Sci..

[29] Heinze T. (2015). Cellulose: Structure and Properties. Adv. Polym. Sci..

[30] Hecht H., Srebnik S. (2016). Structural Characterization of Sodium Alginate and Calcium Alginate. Biomacromolecules.

[31] Mudgil D., Barak S., Khatkar B.S. (2014). Guar Gum: Processing, Properties and Food Applications—A Review. J. Food Sci. Technol..

[32] Cygan R.T., Liang J.J., Kalinichev A.G. (2004). Molecular Models of Hydroxide, Oxyhydroxide, and Clay Phases and the Development of a General Force Field. J. Phys. Chem. B.

[33] Pouvreau M., Greathouse J.A., Cygan R.T., Kalinichev A.G. (2017). Structure of Hydrated Gibbsite and Brucite Edge Surfaces: DFT Results and Further Development of the ClayFF Classical Force Field with Metal-O-H Angle Bending Terms. J. Phys. Chem. C.

[34] Wang J., Wang W., Kollman P.A., Case D.A. (2006). Automatic Atom Type and Bond Type Perception in Molecular Mechanical Calculations. J. Mol. Graph. Model..

[35] Wang J., Wolf R.M., Caldwell J.W., Kollman P.A., Case D.A. (2004). Development and Testing of a General Amber Force Field. J. Comput. Chem..

[36] Li P., Song L.F., Merz K.M. (2015). Systematic Parameterization of Monovalent Ions Employing the Nonbonded Model. J. Chem. Theory Comput..

[37] Berendsen H.J.C., Grigera J.R., Straatsma T.P. (1987). The Missing Term in Effective Pair Potentials. J. Phys. Chem..

[38] Miyamoto S., Kollman P.A. (1992). Settle: An Analytical Version of the SHAKE and RATTLE Algorithm for Rigid Water Models. J. Comput. Chem..

[39] Abraham M.J., Murtola T., Schulz R., Páll S., Smith J.C., Hess B., Lindah E. (2015). Gromacs: High Performance Molecular Simulations through Multi-Level Parallelism from Laptops to Supercomputers. SoftwareX.

[40] Sousa da Silva A.W., Vranken W.F. (2012). ACPYPE—AnteChamber PYthon Parser InterfacE. BMC Res. Notes 2012 51.

[41] Mintis D.G., Alexiou T.S., Mavrantzas V.G. (2020). Effect of PH and Molecular Length on the Structure and Dynamics of Linear and Short-Chain Branched Poly(Ethylene Imine) in Dilute Solution: Scaling Laws from Detailed Molecular Dynamics Simulations. J. Phys. Chem. B.

[42] Mintis D.G., Mavrantzas V.G. (2019). Effect of PH and Molecular Length on the Structure and Dynamics of Short Poly(Acrylic Acid) in Dilute Solution: Detailed Molecular Dynamics Study. J. Phys. Chem. B.

[43] Hess B., Bekker H., Berendsen H.J.C., Fraaije J.G.E.M. (1997). LINCS: A Linear Constraint Solver for Molecular Simulations. J. Comput. Chem..

[44] Hoover W.G. (1985). Canonical Dynamics: Equilibrium Phase-Space Distributions. Phys. Rev. A.

[45] Nosé S. (1984). A Unified Formulation of the Constant Temperature Molecular Dynamics Methods. J. Chem. Phys..

[46] Parrinello M., Rahman A. (1981). Polymorphic Transitions in Single Crystals: A New Molecular Dynamics Method. J. Appl. Phys..

[47] Darden T., York D., Pedersen L. (1993). Particle Mesh Ewald: An N·log(N) Method for Ewald Sums in Large Systems. J. Chem. Phys..

[48] Yoo J., Aksimentiev A. (2018). New Tricks for Old Dogs: Improving the Accuracy of Biomolecular Force Fields by Pair-Specific Corrections to Non-Bonded Interactions. Phys. Chem. Chem. Phys..

